# Preparation, characterization and properties of three different nanomaterials either alone or loaded with nystatin or fluconazole antifungals

**DOI:** 10.1038/s41598-022-26523-1

**Published:** 2022-12-21

**Authors:** Sara H. Helal, Heba M. M. Abdel-Aziz, Mustafa M. El-Zayat, Mohammed N. A. Hasaneen

**Affiliations:** 1 Botany Department, Faculty of Science, Mansoura University, Mansoura, Egypt; 2 Unit of Genetic Engineering and Biotechnology, Faculty of Science, Mansoura University, Mansoura, Egypt

**Keywords:** Biotechnology, Nanobiotechnology, Nanoparticles

## Abstract

Engineered nanoparticles have enabled the development of novel uses, particularly in disease management. In this investigation, we synthesized and studied three distinct nanomaterials: solid lipid nanoparticles (SLNPs), chitosan nanoparticles (CSNPs), and carbon nanotubes (CNTs), either alone or loaded with two antifungals, nystatin, and fluconazole. The purpose of this study is to investigate the different properties of the produced nanomaterials, either alone or in combination with antifungals. Drug release studies revealed that about 55% from SLNPs, 43% from CSNPs and 97% from CNTs of nystatin drug were released at the longest time point assessed (12 h). In addition, about 89% from SLNPs, 84% from CSNPs and 81% from CNTs of fluconazole drug were released at the longest time point assessed (12 h). This research will expand the understanding of nanomaterials as a viable technique for the management of different fungal diseases that harm several agricultural crops.

## Introduction

In recent years, considerable effort has been devoted to the development of nanotechnology for drug delivery, as it provides a suitable method for delivering small molecular weight drugs as well as macromolecules such as proteins, peptides, and genes to cells and tissues while protecting them from enzymatic degradation^1,2^. As medication delivery methods, nanoparticles have the benefits of being biodegradable, non-toxic, and stable enough to be kept for long periods^3,4^.

By manipulating nanotechnology, medications may be loaded onto the surface of nanoparticles or encapsulated and transported within them to their target^5^. In this manner, the effective medication dose can be reduced by several orders of magnitude, hence minimizing adverse effects^6,7^. Recent improvements in plant disease management employing nanoparticles as protectants and as carriers for fungicide, herbicide, and double-stranded RNA for RNA-interference (RNAi)-mediated protection have been made^8^. Nanoparticles are materials ranging in size from 10 to 100 nm (nm), and their chemical, physical, and biological characteristics may be tailored to be distinct from those of their molecule and bulk counterparts^7,9^.

Nanoparticles have the ability to be directly sprayed to plant seeds, leaves, or roots to protect them from insects, bacteria, fungi, and viruses^10^. Nanoparticles are also often employed as carriers to entrap, encapsulate, absorb, or attach active compounds in order to create successful agricultural formulations^8^. Solid lipid nanoparticles (SLNPs) are formed of lipids that are solid at normal temperature and resemble emulsions. A benefit of SLNPs is that they may entrap lipophilic active molecules without the need for organic solvents^11^. Due to the restricted mobility of the active in the solid matrix, SLNPs can also facilitate the regulated release of several lipophilic components^2,11,12^. Surfactants are utilized to stabilize the dispersion of SLNPs in water^13^. Their primary disadvantages are their limited loading efficiency and the possibility of active ingredient leakage during storage^7,14^.

Recently, increasing attention has focused on these SLNPs because as colloid drug carriers they combine advantages of polymeric nanoparticles, fat emulsions, and liposomes simultaneously avoiding some of their disadvantages^15^. Under optimized condotions, SLNPs can be produced to incorporate lipophilic or hydrophilic drugs^15^.

Chitosan is a naturally occurring nontoxic biopolymer derived by deacetylation polymer of N acetyl glucosamine that can be obtained through alkaline deacetylation of chitin. Due to its antibacterial and antifungal activities, chitosan and its derivatives have garnered a considerable amount of attention^5,16–18^. It consists of a B-(1, 4)-linked-d-glucosamine residues with the amine groups randomly acetylated^19^. Chitosan is harmless, non-toxic and can interact with polyanions to create complexes and gels^20,21^. Chitosan is poorly soluble; hence it only has antibacterial effects in acidic media above pH 6.5^22^. Chitosan nanoparticles loaded with antimicrobial agents function by adhering to negatively charged bacterial and fungal cell walls, which causes the cell envelope to become unstable and change its permeability. Next, they adhere to DNA, which prevents it from replicating^5,23–25^. In addition to the positive ionic interactions with the negative charges of the cell surface membranes, the drug can be exposed to microorganisms for a longer period of time^26^.

The antifungal activity of CSNPs made from low molecular weight (LMW) and high molecular weight (HMW) of chitosan has been tested against numerous kinds of fungi. The nanoparticles made with various chitosan concentrations had an inhibitory effect on fungi. The effectiveness of the CSNPs in managing a variety of plant diseases brought on by *Collectotrichum acutatum, Fusarium oxysporum, Rhizoctonia solani, and Phytophthora infestans* was also examined. Moreover, by extending the shelf life of tomato, chili, and brinjal, CSNPs have demonstrated their suitability as a coating agent for coated vegetables. All fungal species were significantly inhibited by CSNPs' antifungal efficacy^27^.

The family of fullerenes (C60) includes carbon nanotubes (CNTs), which are cylindrical sheets of pure carbon that are made of graphite and have nanoscale- and micrometer-sized diameters and lengths^28^. CNTs have different geometrical shapes such as tubes, spherical and ellipsoidal and they may be single walled (SWCNTs), double walled (DWCNTS), or multiwalled (MWCNTS)^29,30^. Furthermore, CNTs are distinguished by their spectacular thermal and electrical properties as well as their great mechanical strength, good chemical stability, extremely low weight, and significant surface area. CNTs' non-immunogenicity and toxicity can be decreased by adding chemical or functional groups to their surface, and this method is crucial for nanomedicine. CNTs can interact with bioactive macromolecules and drugs and transport them to cells and organs^5,31–33^. According to^34^, when bacteria were cultured with CNTs, the quantity and height of the bacterial cells decreased, and the bacterial envelopes were highly affected due to the leakage of intracellular contents. According to^35^, in the pharmaceutical industry, CNTs could be used with antibiotics to the infection transmission site within the body and with a very small dose without toxic effects when compared with the antibiotic alone^7,32^.

In view of the above, the present study aimed to prepare, characterize and compare between three different nanomaterials; solid lipid nanoparticles, chitosan nanoparticles and carbon nanotubes as new and novel nanocarriers for nystatin and fluconazole antifungals.

## Materials and methods

### Preparation of solid lipid nanoparticles (SLNPs) emulsion

A novel and innovative method was adopted for preparation of solid lipid nanoparticles emulsion and loading with either nystatin or fluconazole antifungal antibiotics. Solid lipid nanoparticles as well as loading of nystatin and/or fluconazole on solid lipid nanoparticles were prepared by hot homogenization method with special modification of the method adopted by^36^.

In a glass beaker, 5.0 g of glycerol monostearate lipid were weighted and then melted at 70 ℃ until complete melting. To the melted lipid, 1.0 g of soya lecithin was added (lipophilic surfactant). The mixture was mixed well and then placed in a water bath adjusted at 70 ℃ till complete homogeneity. In another glass beaker, 1.5 cm^3^ of Tween 80 (hydrophilic surfactant) was added and the volume completed up to 100 cm^3^ with distilled water and then the mixture was heated at 70 ℃ for 15 min. with continuous stirring. The lipid phase mixture was added dropwise to the aqueous surfactant mixture. The mixture was homogenized in a graduate high-speed automatic homogenizer at 15,000 rpm for 5 min. The mixture was then sonicated using automatic high speed sonicator for 15 min. The prepared solid lipid nanoparticles (SLNPs) emulsion was stored stable at room temperature until use.

### Loading of the drugs on solid lipid nanoparticles (SLNPs) emulsion

In a glass beaker, 5.0 g of glycerol monostearate lipid and 0.5 g of the drug (nystatin or fluconazole) were weighted and then melted at 70 ℃ until complete melting. Then, the same steps were applied as stated above for preparation of solid lipid nanoparticles (SLNPs). The prepared solid lipid nanoparticles (SLNPs) emulsions loaded with drugs were stored stable at room temperature until use.

### Preparation of chitosan nanoparticles

In the present study, methacrylic acid (MAA) was polymerized in chitosan (CS) solution to prepare chitosan nanoparticles (CSNPs), according to^37,38^. Under magnetic stirring, about 0.2 g of chitosan were dissolved in 0.5 (v/v) of methacrylic acid aqueous solution for 12 h. The prior solution was then heated up at 70 °C and supplemented with 0.005 g of potassium persulfate while being constantly stirred for 1 h until the solution was clear. Finally, the solution was cooled in an ice bath to cease the reaction.

### Loading of the drugs on chitosan nanoparticles

According to^5,38,39^, loading of the antifungal drugs on the surface of CSNPs was performed by adding 10 cm^3^ of the antifungal drug suspension to 20 cm^3^ of CSNPs solution and stirred for 6 h. at room temperature.

### Preparation of carbon nanotubes (CNTs)

CNTs were prepared by using the method of^40^. About 5 g of graphite powder were gradually added to a solution of sulfuric acid and nitric acid (2:1 v/v), stirred for 30 min. and then cooled at 4 °C. Then, 25 g of potassium chlorate was added slowly and gently to the solution and stirred for half an hour followed by heating for 24 h at 70 °C. The floating solution was then rinsed with deionized water to 1000 cm^3^, stirred for l h, filtered, and finally dried.

### Loading of the drugs on CNTs

The loading of CNTs with the antifungal drugs was carried out by adding 10 cm^3^ of the antifungal drugs solution to 50 cm^3^ of CNTs solution and stirring for 6 h at room temperature^41,42^.

## Evaluation studies

The obtained formulations of nystatin and fluconazole loaded nanomaterials were evaluated for the following parameters:

### In vitro drug release kinetics

Nystatin and fluconazole in vitro release patterns from the prepared nanomaterials were determined by a dissolution test in phosphate buffer solution of pH 6.8 and using a regenerated cellulose membrane (12–14 KD molecular weight cut-off). A dialysis bag containing nystatin and fluconazole-loaded nanoparticles was submerged in a 50 cm^3^ phosphate buffer solution, and the system was kept at 37 °C ± 1 under moderate agitation (100 rpm)^43^. The release medium (5 cm^3^) was withdrawn every hour and assayed for drug release and replaced by preheated (37 ℃ ± 1) 5 cm^3^ of fresh buffer (pH 6.8 phosphate buffer) for continuous 12 h. By comparing the amount of nystatin and fluconazole in the release medium to a blank and measuring them using UV spectrophotometer at 322 nm and 260 nm, respectively, the cumulative drug levels of nystatin and fluconazole were estimated. Cumulative drug of nystatin and fluconazole were calculated based on a pre-made calibration curve.

### Characterization of nanomaterials either alone or loaded with nystatin and fluconazole

#### Physical characterization

##### Morphology and size

Transmission electron microscope (TEM), (JEOL 1010, EM Unit, Mansoura University, Egypt), micrographs were taken to investigate the morphology and size of the prepared nanomaterials either alone or loaded with nystatin and fluconazole.

##### Electron diffraction pattern for prepared nanomaterials

Electron diffraction is the most direct and fast technique that gives access to detailed information about the structure of nanomaterials either alone or loaded with nystatin and fluconazole^44^ and it was performed using transmission electron microscope (TEM) (JEOL 1010, EM Unit, Mansoura University, Egypt).

#### Chemical characteristics

##### Measurement of zeta potential

Zeta potential values of the prepared nanomaterials alone or loaded with antifungals were measured on zeta-sizer (Malvern Instruments ltd, EM unit, Mansoura University, Mansoura, Egypt). Zeta cell was cleaned with distilled water, ethyl alcohol, and then again with distilled water before being dried with a moderate nitrogen stream to remove any remaining solvent. It was then covered to avoid contamination. Three runs for each sample were carried out after about l cm^3^ of sample was carefully injected into the cell using a syringe^45^.

#### FTIR analysis

The FTIR measurements for single or loaded nanomaterials were performed using the method of^46^. A mixture of 0.1 g of potassium bromide (spectrally pure) and about 0.003 g of sample was compressed for 10 min under vacuum to create a greyish circular disc, which was then analyzed using a Fourier transform spectrometer. (NICOLET IS10 FT-IR instrument, Faculty of Science, Mansoura University).

### Statistical analysis

All the test experiments were recorded in triplicates. The results were performed in the form of mean values. In addition, the results were statistically analyzed using one way ANOVA by software system SPSS version 18 with statistically significant differences at * *p* values ≤ 0.05.

## Results

### Characterization of prepared nanomaterials either singly or loaded with nystatin and fluconazole antifungal antibiotics

#### Physical characterization

##### Morphology and size

**Figure 1:**
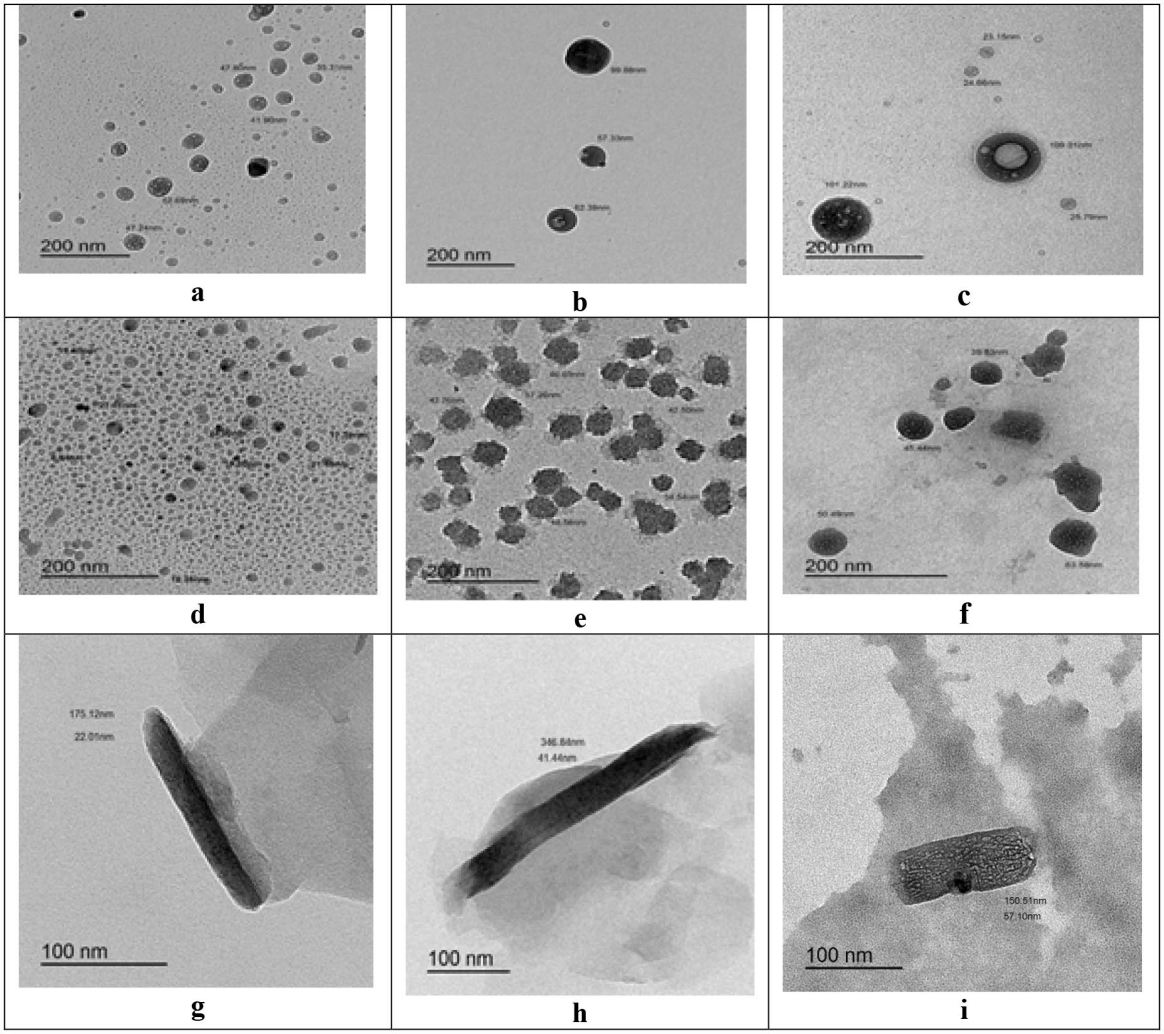
TEM micrograph revealing (**a**) SLNPs, (**b**) SLNPs loaded with nystatin, (**c**) SLNPs loaded with fluconazole, (**d**) chitosan nanoparticles (CSNPs), (**e**) chitosan nanoparticles loaded with nystatin, (**f**) chitosan nanoparticles loaded with fluconazole, (**g**) a multiwalled carbon nanotube with a diameter of 22.01 nm, (**h**) a carbon nanotube (CNTs) loaded with nystatin with a diameter of 41.44 nm and (**i**) a carbon nanotube (CNTs) loaded with fluconazole with a diameter of 57.10 nm.

As shown in Fig. 1, TEM examination show the CSNPs are roughly round in form and display a highly compact structure with a diameter range 8–29 nm. Careful examination of Fig. 1 reveals that the addition of both antifungal drugs; nystatin and fluconazole to complex nanoparticles led to an increase in the sizes of nanoparticles. The range of increase in the diameter of nanoparticles was 34–57 nm for nystatin (Fig. 1) and 39–63 nm for fluconazole (Fig. 1). The loading of nystatin resulted in a maximum increase in mean diameter of 96.55%, while the loading of fluconazole led to a maximum increase of 117.24% compared to the diameter of nanomaterial alone.

In the present study, transmission electron microscopy (TEM) images were taken to characterize the morphology of the resulting carbon nanotube minutely. Figure 1 shows TEM micrograph of the prepared carbon nanotubes which demonstrated long and stripe-like carbon nanotube (CNTs) with diameter of 22.01 nm for the single tube. Loading of nystatin and fluconazole antifungal antibiotics led to increase in diameter of the loaded tubes (Fig. 1). Figure 1 emphasizes that increase of tube diameter up to 41.44 nm for nystatin and up to 57.10 nm in case of fluconazole with maximum increases in mean diameter of 100% and 157.20%, respectively.

Figure 1 shows transmission electron micrographs of SLNPs either alone or loaded with nystatin and fluconazole antifungals. The tested pictures demonstrated round and homogenous shape of the nanoparticles; the figures also confirmed that the prepared SLNPs were less than 100 nm and exactly ranged from 32 to 52 nm singly and 57–99 nm loaded with nystatin and 62–109 nm loaded with fluconazole antifungals.

The results herein indicated that loading of nystatin and fluconazole on SLNPs resulted in a remarkable increase in the diameter. Figure 1 shows TEM micrographs of solid lipid nanoparticles loaded with nystatin and fluconazole antifungals that detect the size and the morphology of the prepared nanomaterials. Careful examination of Fig. 1 reveals that the loading of nystatin and fluconazole on solid lipid nanoparticles led to increase the sizes of SLNPs. The maximum increase in the mean diameter was 90.38% with the addition of nystatin and 109.62% with the addition of fluconazole.

##### Structure of SLNPs, CSNPs and CNTs electron diffraction (ED)

**Figure 2:**
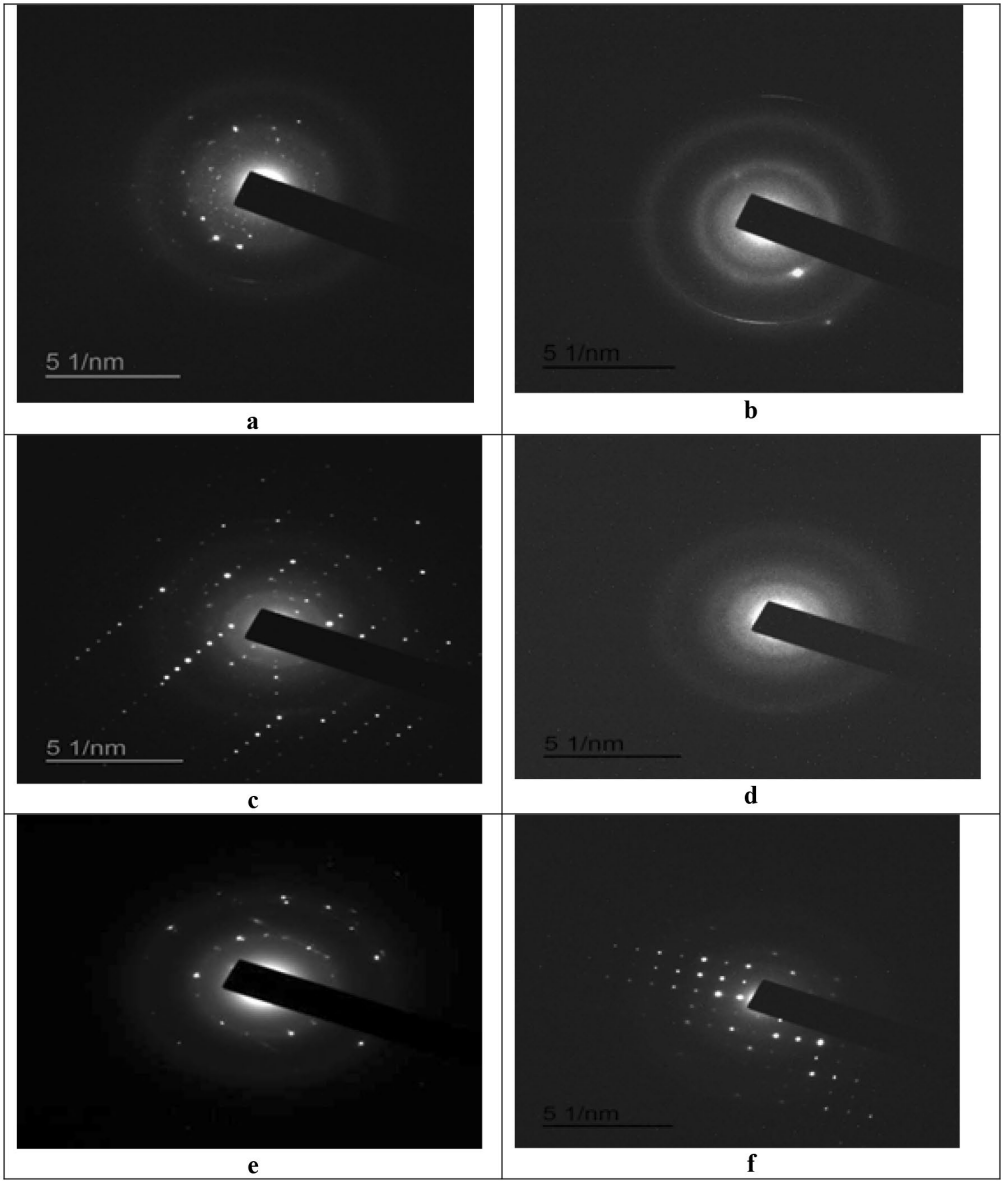
Electron diffraction pattern of (**a**) SLNPs loaded with nystatin, (**b**) SLNPs loaded with fluconazole, (**c**) chitosan nanoparticles loaded with nystatin, (**d**) chitosan nanoparticles loaded with fluconazole, (**e**) carbon nanotubes loaded with nystatin and (**f**) carbon nanotubes loaded with fluconazole.

To determine the structure of SLNPs loaded with nystatin and fluconazole, electron diffraction was performed. Figure 2 show an electron image indicates that the SLNPs loaded with nystatin and fluconazole have zigzag edges. The electron diffraction patterns indicate rotational crystal pattern. To determine the structure of CSNPs loaded with nystatin and fluconazole, electron diffraction was performed. Figure 2 show an electron image indicates that the CSNPs loaded with nystatin and fluconazole have zigzag edges. The diffraction pattern looks like rotation crystal pattern. Ring and spot pattern appear together implying that the spherical CSNPs are comprised of both crystalline amorphous phases. To determine the structure of CNTs loaded with nystatin and fluconazole, the electron diffraction pattern indicates that the tube has nearly identical chirality for all of the concentric graphitic layers, as a zigzag-type CNTs. Figure 2 show that the diffraction configurations exhibit rotational crystal patterns. Rings and spots indicating that the nanotubes contain zigzag edges and are crystallized.

#### Chemical characterization

##### Zeta potential

Zeta potential is an important factor in physical stability of nanoparticles. The higher zeta potential value shows better stability of the dispersion. Except for the observed positive zeta potential values of all CSNPs solutions, zeta potential values of all other formulations were with negative values (Table 1).

**Table 1:** Average zeta potential values of the prepared nanomaterials either alone or loaded with nystatin and fluconazole.

Nanosuspension	ζ -Potential (mV)
SLNPs	− 21.30
SLNPs-NYS	− 23.50
SLNPs-FLZ	− 25.20
CSNPs	+ 25.40
CSNPs-NYS	+ 49.20
CSNPs-FLZ	+ 32.70
CNTs	− 8.65
CNTs-NYS	− 9.39
CNTs-FLZ	− 9.73

##### Fourier transformation infrared spectroscopy (FTIR)

Supplementary figures show FTIR spectra for all the prepared nanomaterials either alone or loaded with antifungals. Figure S1 shows FTIR spectrum of GMS (lipid core and main component of SLNPs), which shows absorption peak at 3392 cm^−1^ that represents the stretching vibration of the hydroxyl group (–OH) and 2916 cm^−1^ and 2850 cm^−1^ were ascribed to (–CH_3_) and –(CH_2_) stretching vibrations, respectively. The peak at approximately 1735 cm^−1^ was the stretching vibration of the ester group and 1000–1300 cm^−1^ was the stretching vibration of C–O stretching. Figure S2 emphasizes the presence of the characteristic broad peak of nystatin drug loaded on SLNPs at about 3388 cm^−1^ region that was characteristic of overlapping between N–H and O–H stretching vibrations.

As shown in Fig. S3, the fluconazole loaded SLNPs spectrum showed characteristic peaks at around 3381 cm^−1^ (–OH stretching vibrations). This spectrum overlapped with GMS spectrum in some positions. These FTIR results clearly revealed entrapment of the two antifungal drugs in lipid matrix and proved no chemical interaction between drugs and its carrier.

FTIR spectra of the prepared CSNPs nanoparticles presented in Fig. S4 show the presence of two characteristic absorption peaks at 1638 and 1545 cm^−1^ corresponding to COO^−^ and NH^+3^ groups, respectively, which indicate ionic interaction between PMAA and CS related to the formation of nanoparticles. The bands at 1703 and 1264 cm^−1^ (C=O) confirm the presence of PMAA in the nanoparticle composition. Figure S5 emphasizes the presence of the characteristic broad peak of nystatin drug loaded on CSNPs at about 3388 cm^−1^ region that was characteristic of overlapping between N–H and O–H stretching vibrations. Figure S6 showed the fluconazole loaded CSNPs spectrum showed characteristic peaks at around 3381 cm^−1^ (–OH stretching vibrations).

In the measured IR absorbance spectrum (Fig. S7), peaks at 2915 and 2854 cm^−1^ are caused by C–H vibrations of alkyl group which are a residue of hydrocarbon molecules used for growing the CNTs. The band at 3448 cm^−1^ can be attributed to vibrations of O–H bonds in hydroxyl and carboxyl groups formed upon the oxidation of the nanotubes, the absorption peaks at 1573 and 1634 cm^−1^ can be attributed to > C=O groups. IR spectrum of the CNTs oxidized with nitric acid is characterized by the presence of absorption bands corresponding to C–H (2923, 2854 and 1462 cm^-1^), >C=C< 1636 cm^−1^, and O–H (3450 cm^−1^) bonds. Figures S8 and S9 show the characteristic bands for loading of nystatin and fluconazole on CNTs.

### In vitro release study

Dialysis bag method was used for nystatin and fluconazole study. Dialysis tube was soaked in the release medium overnight. To ensure sink conditions, tween 80 was added to release medium. Membrane diffusion techniques are widely used for the study of drug in vitro release incorporated in colloidal system. In these cases, drug release follows more than one mechanism. In case of release from the surface of the SLNPs, adsorbed drug quickly dissolved when it comes in contact with the release medium. Drug release by diffusion involves these steps. Briefly, water penetrate into system and causes swelling of mixture followed by the conversion of solid lipid into rubbery matrix and then the diffusion of drug from the swollen rubbery matrix takes place.

**Figure 3:**
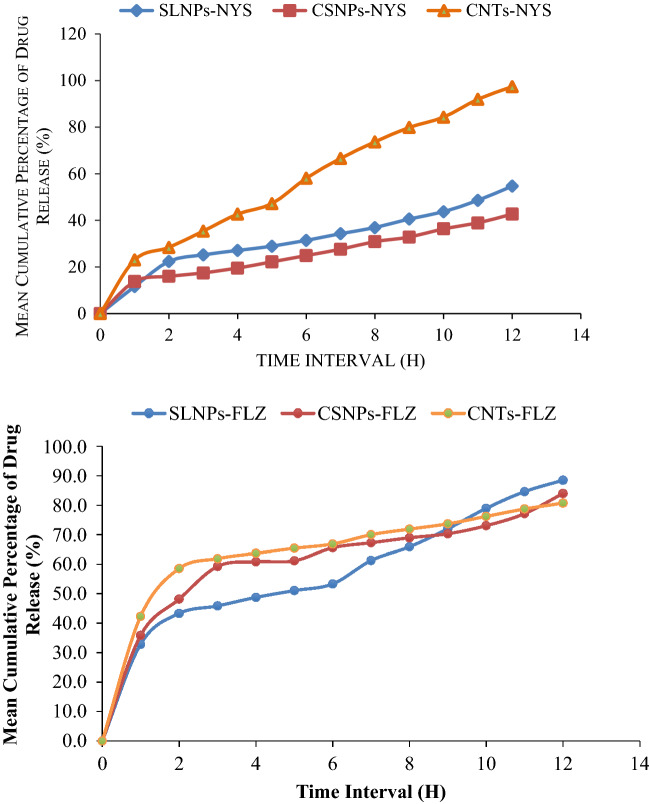
Drug release of nystatin or fluconazole from the prepared nanomaterials.

The encapsulation efficiency of nystatin in solid lipid nanoparticles, chitosan nanoparticles and carbon nanotubes were 29%, 22% and 58% respectively, and the drug was released in sustained manner from nystatin-based nanoparticles approximately 29%, 22% and 58% of the drug was released in the first 6 h (Fig. 3). Furthermore, about 55% from SLNPs, 43% from CSNPs and 97% from CNTs of nystatin drug were released at the longest time point assessed (12 h).

On the other hand, perusal of data presented in Fig. 3 revealed that the release rate of fluconazole drug from SLNPs, CSNPs and CNTs were approximately 53%, 61% and 65% respectively, and the drug was released in sustained manner from fluconazole-based nanoparticles approximately 53%, 61% and 65% for SLNPs, CSNPs and CNTs, respectively, of the drug was released in the first 6 h (Fig. 3). In addition, about 89% from SLNPs, 84% from CSNPs and 81% from CNTs of fluconazole drug were released at the longest time point assessed (12 h).

## Discussion

The current study focuses on the creation of experimental trials to find nanomaterial systems that allow us to improve and restore the antibiotic action for organisms that are resistant to drugs. In order to combat the ineffectiveness of traditional antibiotics and get around treatment restrictions associated with these illnesses, new and more aggressive antibiotic resistant bacteria, fungi, and parasites necessitate the development of novel therapeutic techniques. In particular, nanostructured biomaterials, or nanoparticles, have special physicochemical characteristics, such as extremely small and controllable size, a high surface area to mass ratio, high reactivity, and functionalized structure.

The greater adsorption capacity of CNTs, SLNPs, and CSNPs due to their hollow and porous structure, their hydrophobic and hydrophilic surfaces, their enormous surface area, and the strong interaction between antibiotic molecules and SLNPs, CNTs, and CSNPs may be responsible for the TEM results reported in this study^47,48^. Since the generated nanoparticles in this study are less than 150 nm, they are stable for transport in living cells. Nanoparticle size plays a significant influence in the physical stability and uptake of nanocarriers^49^. The CSNPs, CNTs, and SLNPs created with nystatin and fluconazole loaded are smaller than those created by previous studies^50^. Due to Brownian motion, these smaller particles move faster, resulting in dispersion stability against gravity^51^.

CSNPs have a much more content of primary amino acids and hydroxyl groups which are incorporated with ions or molecules by simple chelation or by ion exchanges forming various chemical bonds with these ions which enhances the stability of nanoparticles and prevent agglomeration^52^. In support of these results^41^, reported that chitosan nanoparticles size was increased by 53% with the addition of 60 ppm of phosphorus, 32% with the addition of 400 ppm of nitrogen, and 13% with the addition of 400 ppm of potassium.

It was shown that the mean diameter of the prepared CNTs was of approximately 17.92 nm and increased with the addition of the different compounds such as urea, calcium phosphate and potassium chloride as a source of NPK^42^. This attributed to the greater adsorption capacity of CNTs due to their hollow and porous structure, their hydrophobic surface, huge surface area, and strong interaction between fertilizer molecules and CNTs^47,48^. This adsorption ability is attributed to the presence of high energy adsorption sites like functional groups. CNTs defects and groove interstitial regions between CNTs bundles^47,48^.

As already mentioned, electron diffraction is the most direct fast technique that gives access to detailed information about the structures of nanomaterials (CNTs, CSNPs and SLNPs)^44^. Electron diffraction pattern in the present study (see Fig. 2) indicated that the tubes of CNTs, spherical round shape of SLNPs and spherical shaped CSNPs have nearly identical chirality as a zigzag-type. The diffraction configuration exhibit rotational crystal patterns. From Fig. 2, the appeared rings and spots indicated that loaded nanomaterials contain zigzag edges and are crystallized^42^.

It was stated that to determine the type of the prepared nanomaterial, electron diffraction was performed^42^. Electron diffraction refers to the wave nature of electrons and form a different pattern; it is possible to reveal the exact atomic structure of individual nanomaterials. As shown in Fig. 2, the appeared spots demonstrate that nanomaterials contain zigzag edges and are crystallized^5^.

The zeta potential of CSNPs, whether used alone or in combination with nystatin and fluconazole, had varying positive values, as indicated in table (1). Due to the cationic properties of chitosan, these positive values showed that the CSNPs are positively loaded, and high values suggested that the nanoparticles are stable. Nystatin and fluconazole loading alters the zeta potential values, which shows that the surface of the CSNPs has been loaded with additional charges^53^. CNTs, on the other hand, have negative zeta potential values, as displayed in Table 1. As a result, they were shown to be negatively loaded, and the solutions' low negative values made it clear that they are unstable.

Nystatin and fluconazole antifungals are added, and the zeta potential levels are negatively shifted. Additional charges on the surface of carbon nanotubes are thought to be the cause of this rise^37^. Table 1 shows that all SLNPs formulations had an average zeta potential that was negative, ranging from − 21.3 mV for SLN to − 23.5 mV and − 25.2 mV for SLNPs loaded with nystatin and fluconazole, respectively (see Table 1). Lecithin is mostly responsible for the negative charge, although tween 80's hydrogels can also provide a very tiny negative charge^54^. A minimum zeta potential of − 21.3 mV is required for this study's nanosuspension stabilisation because lecithin and tween 80 work together to combine electrostatic and steric forces, respectively^14,54^. The zeta potential of nanocarriers considerably enhanced as a result of nystatin and fluconazole loading in the SLNPs (see Table 1). Furthermore, in the absence of other elements like steric stabilisers or hydrophilic surface appendages, larger values of zeta potential may cause particles to disaggregate^55^. Zeta potential measurements enable predictions regarding colloidal dispersion storage stability.

It was reported that CNTs were negatively surface charged depending on the media used^42^. It is obvious that the CNTs, CNTs-N, CNTs-P, and CNTs-K solutions were more sensitive to charge in the reaction pH leading to a variation in the zeta potential values because of the charge in energy electric field in the CNTs surface surrounded by other ions^42^.

It was observed that the zeta potential of CS-PMAA nanoparticles either alone or as a function of interaction of N, P, and K at different pH values had positive values^38^. These positive values of the zeta potential indicate that CS-PMAA nanoparticles are positively loaded due to the cationic features of chitosan and high values implying that the solutions were stable. The variation of the zeta potential values with each of CS-PMAAN, CS-PMAAP, and CS-PMAAK composite indicate the loading of more charges on the CS-PMAA nanoparticles surface^38^.

Infrared spectroscopy is a non-destructive analysis method that was used to find interactions between materials that might happen during the creation of nanoparticles^56^. The FTIR spectra of several of the created nanocarriers as well as pure lipids from SLNPs is presented in the supplemental figures. Each spectrum of SLNPs loaded nanocarriers was compared with SLNPs bulk nanocarriers in order to examine the interaction of stearin lipid with other components that are used for the manufacturing of nanocarriers. The vibration bands of (–OH) at 3392 cm^− 1^ and (–CH_3_) and –(CH_2_) at 2916 cm^−1^ and 2850 cm^−1^, respectively, were visible in the FTIR spectrum of SLNPs^57^.

The absence of new bands of nystatin or fluconazole SLNPs indicated that there was no chemical reaction between the drug and lipid matrix, being only dissolved in lipid matrix of glycerol monostearate (GMS). These results were in full agreement with that obtained from ref who studied that FTIR of erythromycin loaded on different SLN formulations. In FTIR spectrum (supplemental figures), the characteristic bands observed from the data of SLNPs singly or loaded with nystatin and fluconazole drugs included the –OH group in the range 3600–3200 cm^−1^, C–H stretching in the range of 3000 cm^−1^ and 2900 cm^−1^, C=O in 1755–1650 cm^−1^, C=C in 1690–1635 cm^−1^ and C–O–C in 1300–1000 cm^−1^^58^.

Depending on the synthetic procedure CSNPs, CNTs and SLNPs may contain various functional groups such as –NH4, N–N, C=O, –OH and –COOH. These functional groups can be added by oxidation or removed by heat treatment^5^. It was stated that the peak at 1534 cm^−1^ implies that the CNTs regions consist of sp^2^ bonded carbon^38^. Peaks at 2915 cm^−1^ and 2854 cm^−1^ are caused by C-H vibrations of alkyl group which are hydrocarbon molecules residue formed during the growing of CNTs.

The most prevalent mechanism explaining how chitosan has antifungal and antibacterial activities is that it binds to negatively charged bacterial surfaces, disrupting and changing the permeability of bacterial cell membranes. This permits materials to leak out of the bacterial and fungal cells resulting in cell death^59^. Chitosan is a mucoadhesive polymer that can break down tight junctions, enabling the administration of vaccinations by paracellular transport of molecules across mucosa^60–62^.

When compared to conventional carriers, the large surface area of CNTs allows for a higher loading capacity of various drug types and improved drug delivery. Due to their enhanced permeability and solid tumor retention function, CNTs loaded with therapeutic compounds can eventually reach the tumor tissues and microbial pathogens^32,63–65^. The cytotoxicity produced by CNTs is influenced by a variety of factors, including adsorption, functionalization level, CNT size and length, cell line, type of tissue and material, degree and type of accumulation, and application method^66^.

Techniques for membrane diffusion are frequently employed to explore medication in vitro release in colloidal systems. In this instance, there are multiple mechanisms used for medication release. When a drug is adsorbed onto CSNPs, CNTs, or SLNPs and then released from their surface, the drug dissolves quickly when it comes into touch with the release medium. These processes are involved in drug release by diffusion. In a nutshell, water infiltrates the system and produces matrix swelling. Next, nanomaterials are converted into rubbery matrix, and last, drug diffusion occurs from the rubbery matrix's swollen state. Consequently, the release starts out slowly before becoming quick^20^.

A study investigated the antibacterial activity of ciprofloxacin loaded on MWCNTs-gelatin-chitosan nanocomposite and assessed how MWCNTs affected the rate of drug release^67^. After an hour, there was a sudden release of the drug, but it was controlled by a slow decline in pace. The antibacterial activity produced by the drug integrated into the MWCNTs-gelatin-chitosan nanocomposite and the drug inserted into the gelatin-chitosan composite without MWCNTs were found to differ significantly. For all studied microorganisms, the antibacterial activity of drug loaded nanocomposite were found to be superior to those of drug loaded on gelatin-chitosan composite without MWCNTs^67^.

One of the appealing features of their utilisation is the prolonged release of medications, which is supported by SLNPs-based nanocarriers and may enable the drug to be continuously and gradually delivered into the body. The stearate polymer is hydrophilic, and many routes for drug release have been described: poorly water-soluble medications appear to be released predominantly by matrix erosion, as opposed to water-soluble pharmaceuticals^68^. A study noted a burst of nystatin release from alginate microparticles in the first stage, followed by a slower persistent release phase^69^. For MFS from the alginate nanoparticles, a similar pattern of behaviour was seen, with a higher release of the drug shown at 6 h, followed by a slower and steadier condition for up to 24 h^70^. There shouldn't be any significant formulation-related barriers to medication release because FTIR analysis found no chemical interactions between the medicines and the nanocarriers. The observed delayed release could result in less frequent dosage and the likelihood of negative effects.

## Conclusion

The current study focuses on the creation of experimental trials to find nanomaterial systems that allow us to improve and restore the antifungal action for organisms that are resistant to drugs. In particular, nanostructured biomaterials, or nanoparticles, have special physicochemical characteristics, such as extremely small and controllable size, a high surface area to mass ratio, high reactivity, functionalized structure, and physico-chemical properties. In this context, we were able to prepare and characterize three different nanomaterials: SLNPs, CSNPs, and CNTs either alone or loaded with two antifungals: nystatin or fluconazole. Based on the rate of drug release from the surface of nanomaterials, our results show clearly that the following sequence: SLNPs-Flu > SLNPs-Nys > CSNPs-Flu > CSNPs-Nys > CNTs-Nys > CNTs-Flu was displayed with respect to ideal application as nanodrug deliver strategy. This study is promising and opens up a new strategy for developing new ways to control drug release of antifungals. Further in vitro and in vivo studies will be applied to test the effectiveness of the produced nanomaterials on tested fungi, therefore, bioconjugation and encapsulation of nanoparticles with bioactive molecules is promising field which minimize the risk of toxicity. Hence in order to gain successful usage and commercialization of nanomaterials, different expertise should collaborate to design bio-mimetic nanomaterials and their evaluation in agriculture sector.

## Supplementary Information

Supplementary Figures.

## Data Availability

All data generated or analyzed during this study are included in this article.
