# Supplementary material for: Preparation, characterization and properties of three different nanomaterials either alone or loaded with nystatin or fluconazole antifungals
Source: Sci Rep. 2022 Dec 21;12:22110. doi: 10.1038/s41598-022-26523-1 (PMC9772394; doi:10.1038/s41598-022-26523-1)
Supplement: Supplementary file 1 — Supplementary Figures. [file 41598_2022_26523_MOESM1_ESM.pdf]

Preparation, characterization and properties of three different nanomaterials either alone or loaded with Nystatin or Fluconazole antifungals

Sara H. Helal[1], Heba M. M. Abdel-Aziz*[,1], Mustafa M. El-Zayat[2] and Mohammed N. A. Hasaneen[1]

[1]Botany Department, Faculty of Science, Mansoura University, Mansoura, Egypt.

[2]Unit of Genetic Engineering and Biotechnology, Faculty of Science, Mansoura University, Egypt

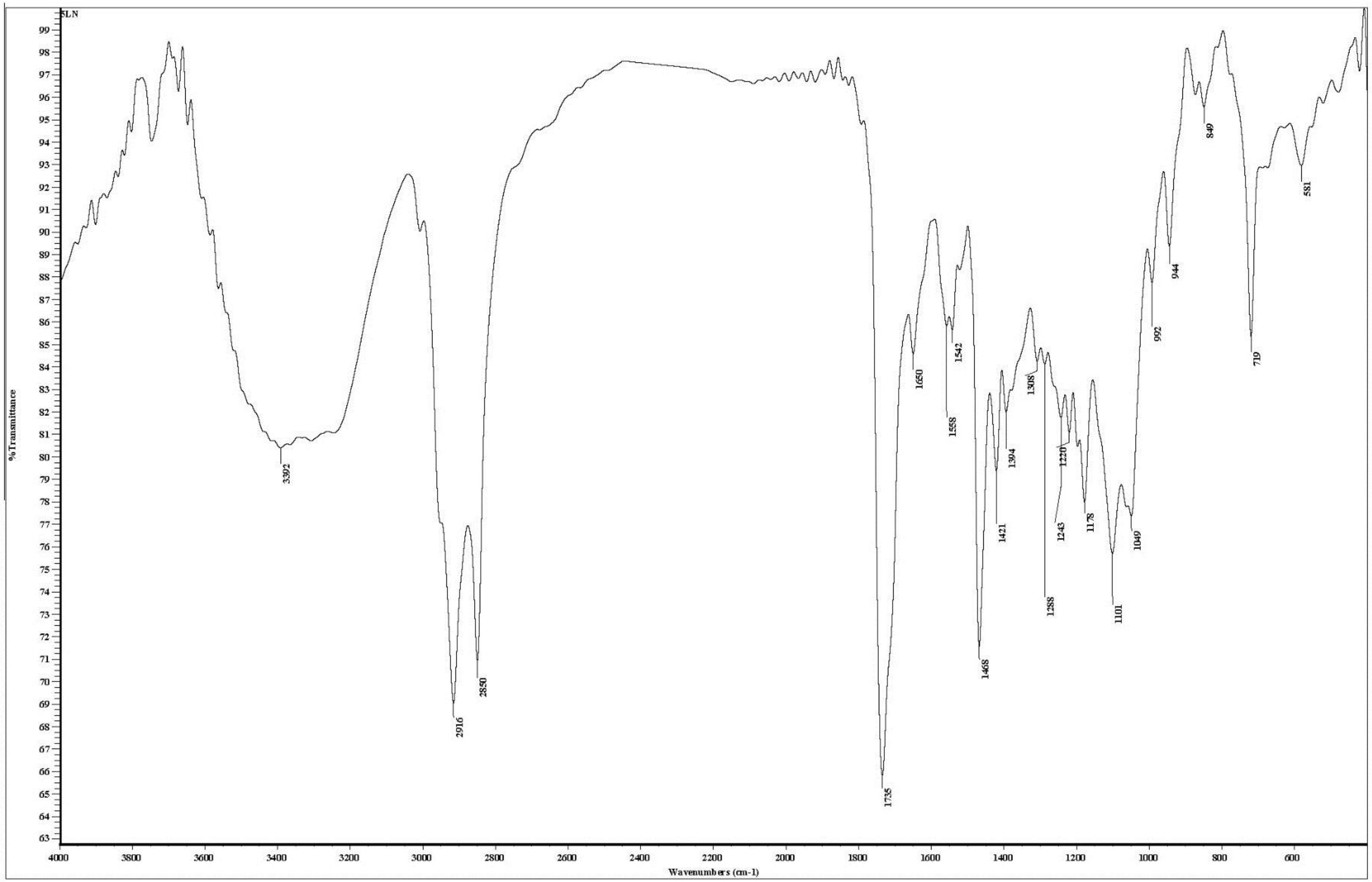

Figure S1. FTIR spectrum for pure solid lipid nanoparticles (SLNPs).

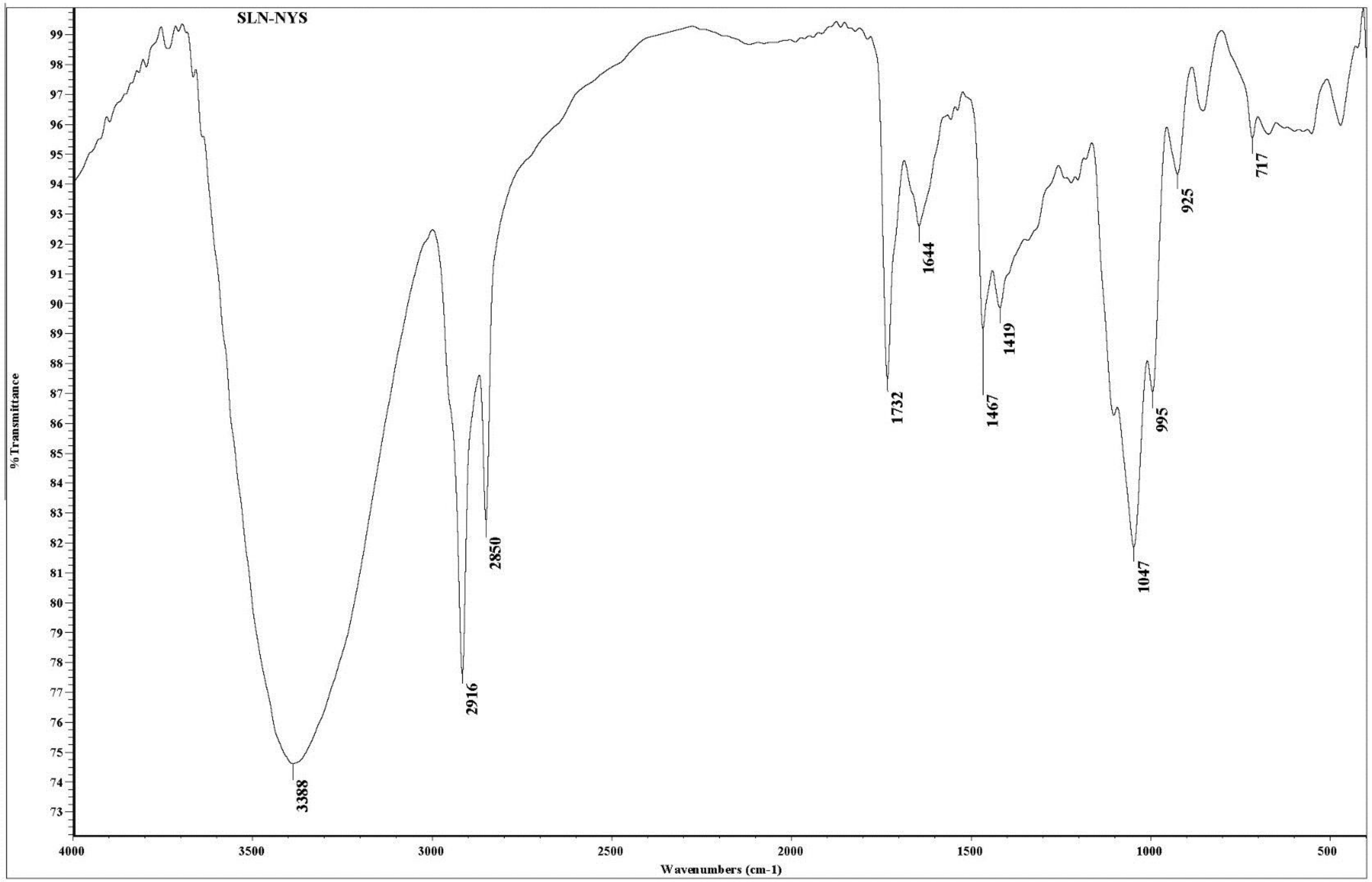

Figure S2. FTIR spectrum for SLNPs loaded with nystatin.

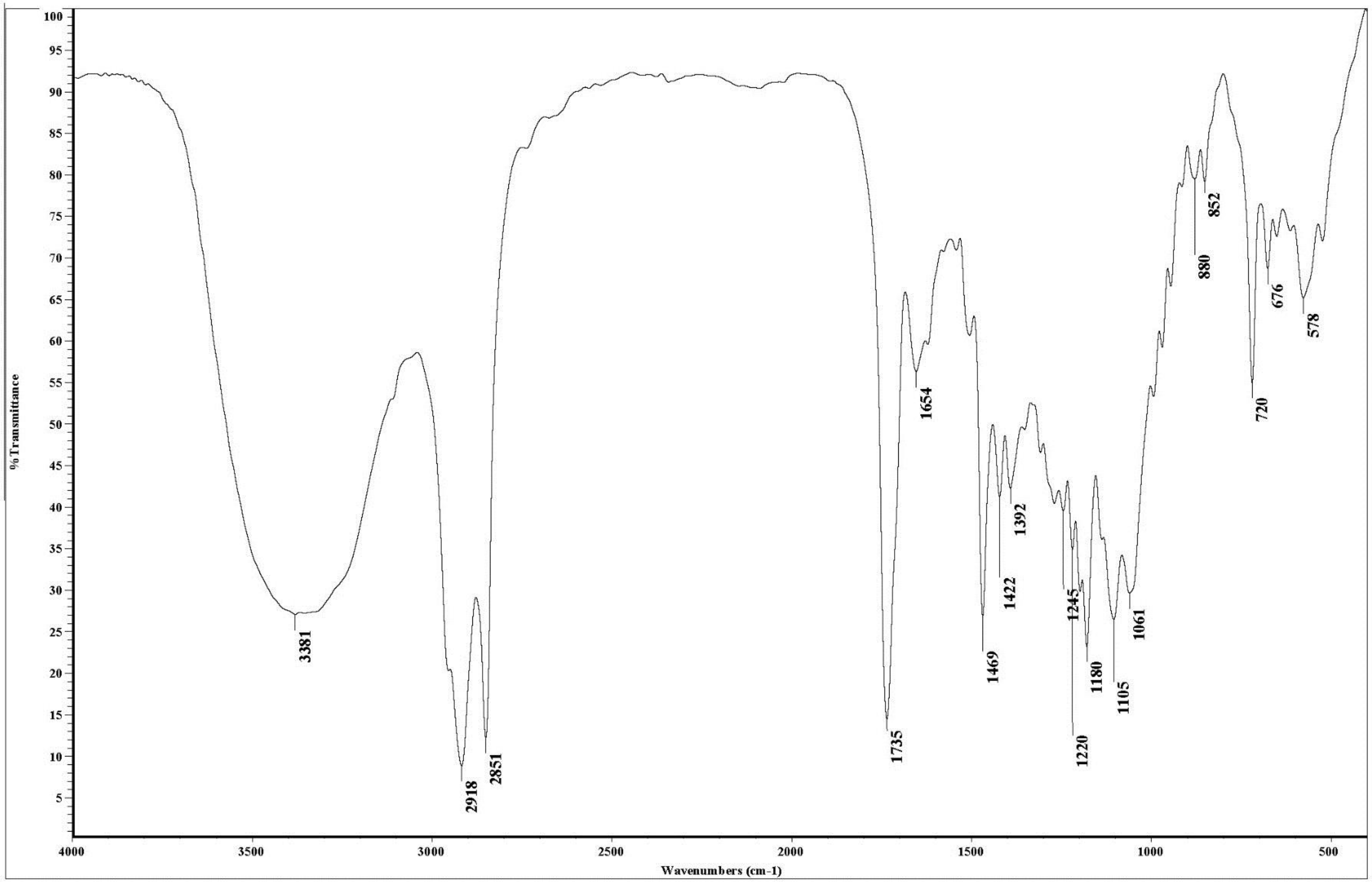

Figure S3. FTIR spectrum for SLNPs loaded with fluconazole.

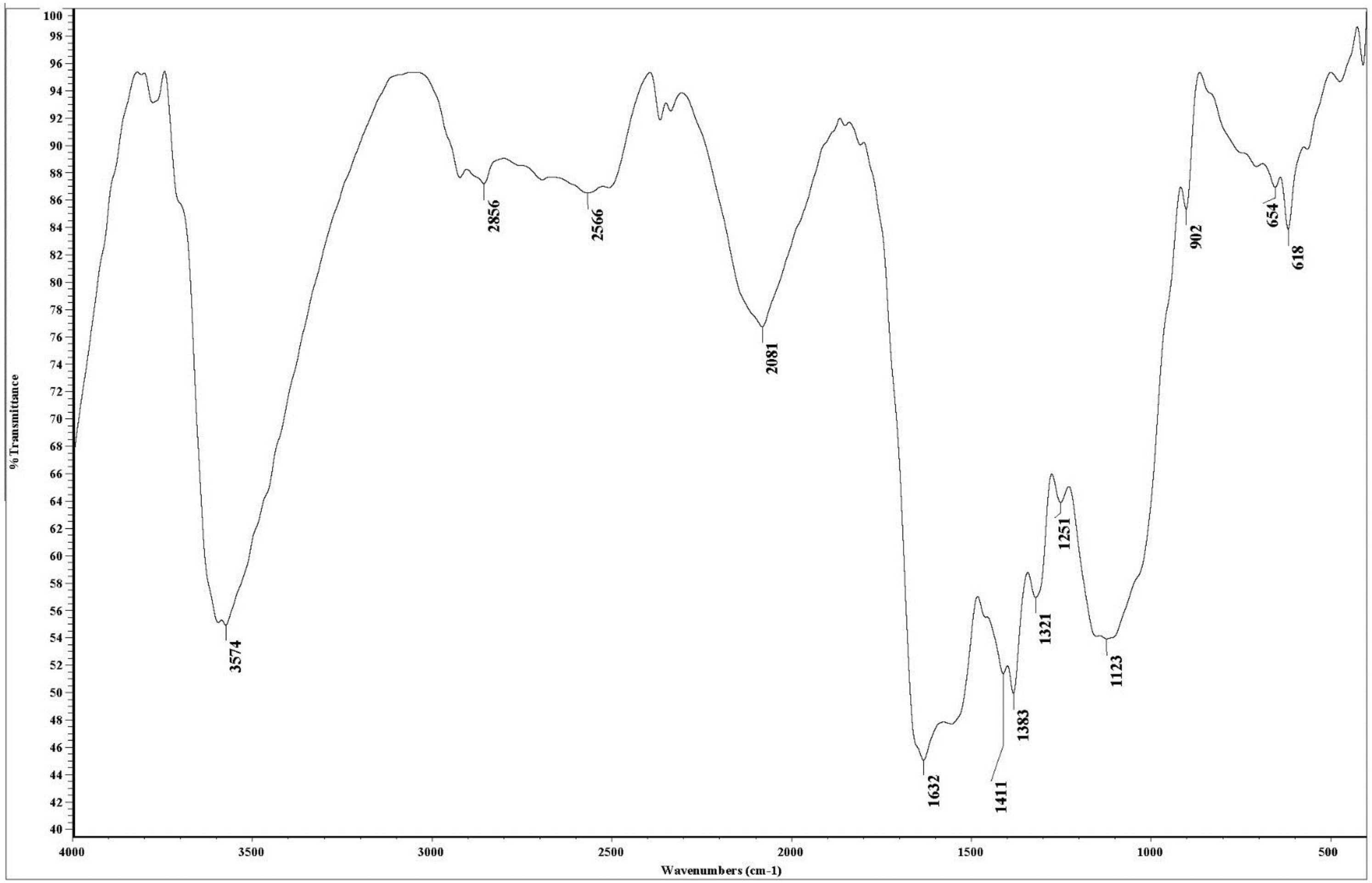

Figure S4. FTIR spectrum for pure chitosan nanoparticle (CSNPs).

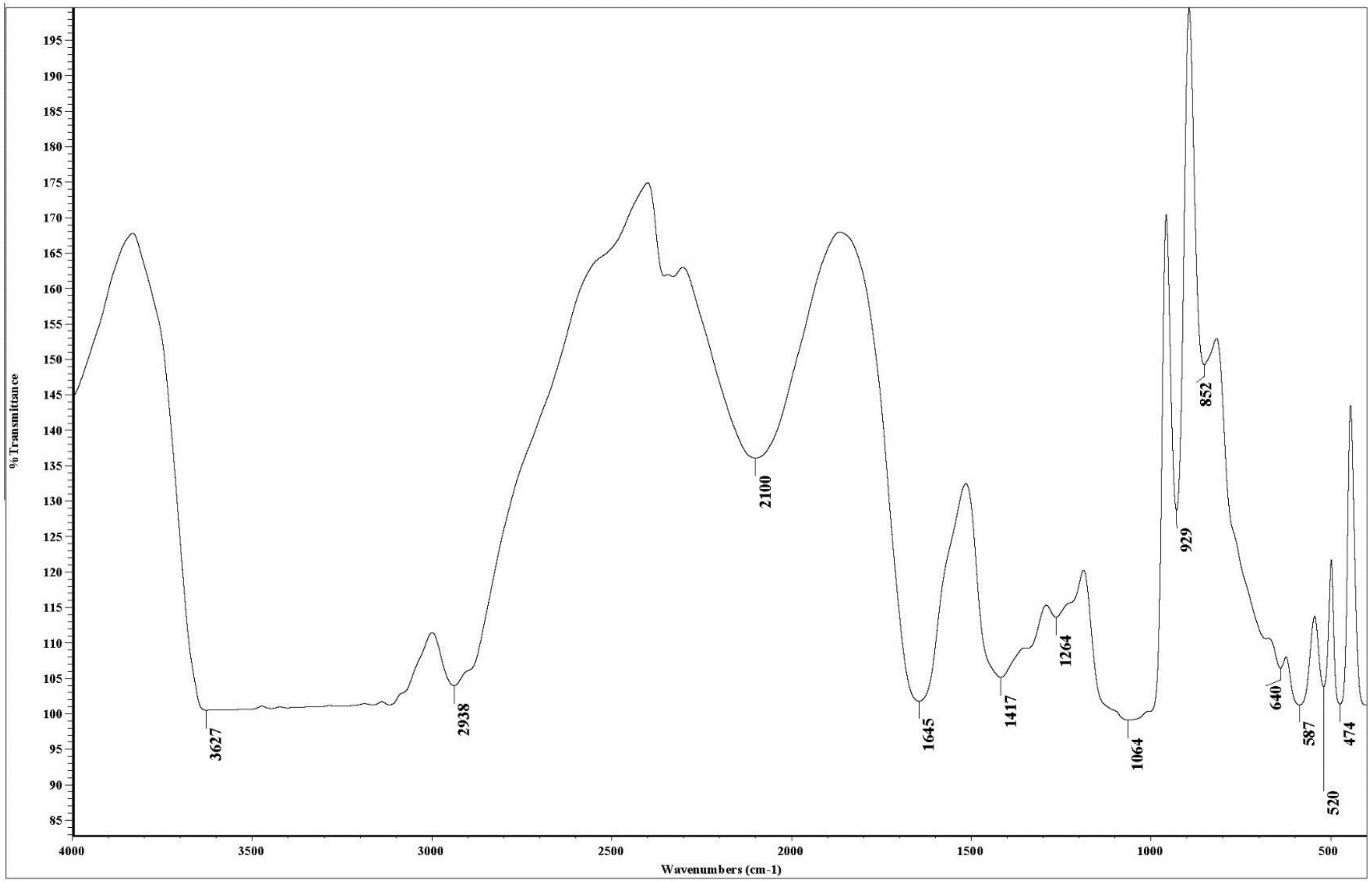

Figure S5. FTIR spectrum for CSNPs loaded with nystatin.

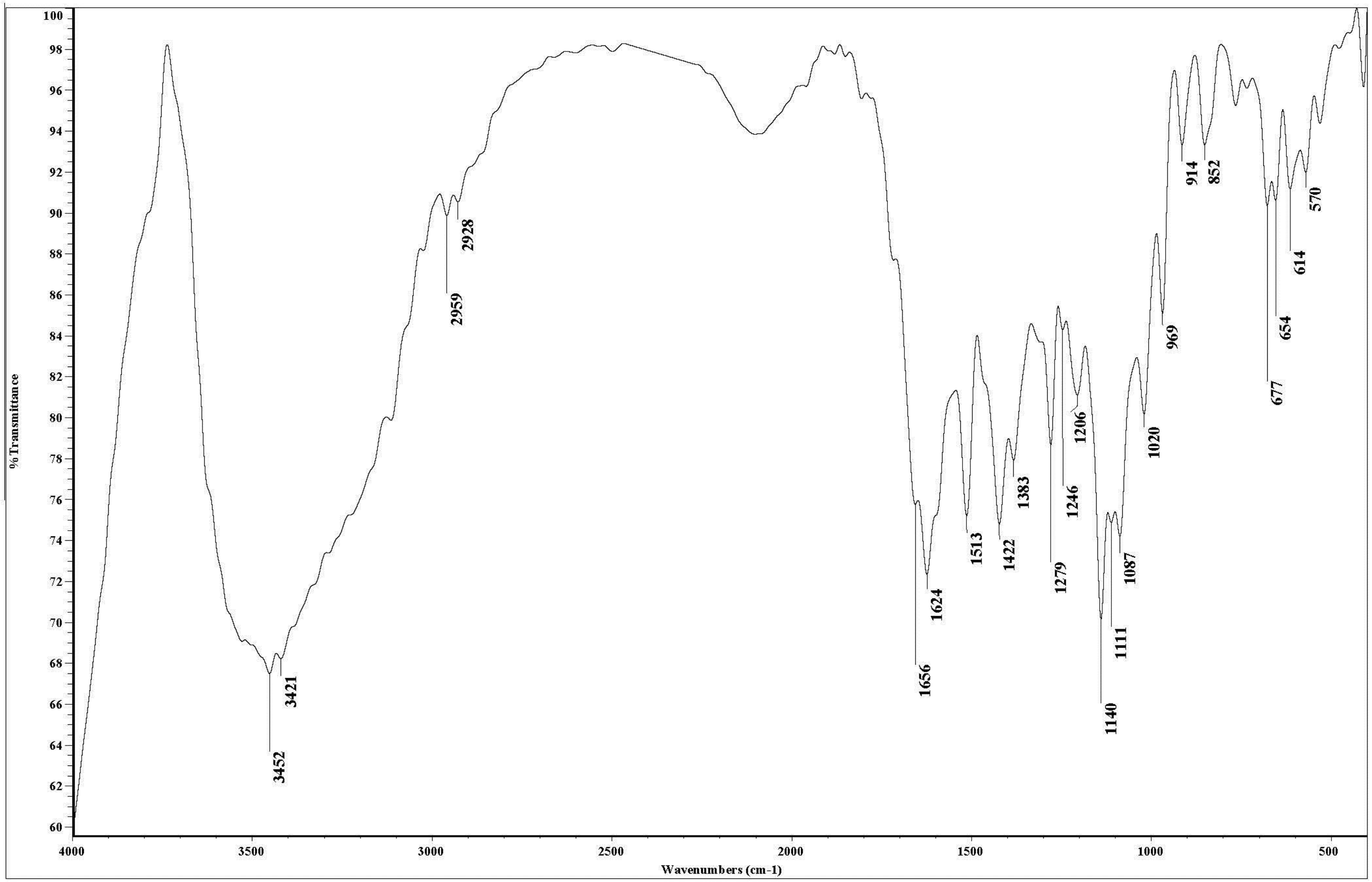

Figure S6. FTIR spectrum for CSNPs loaded with fluconazole.

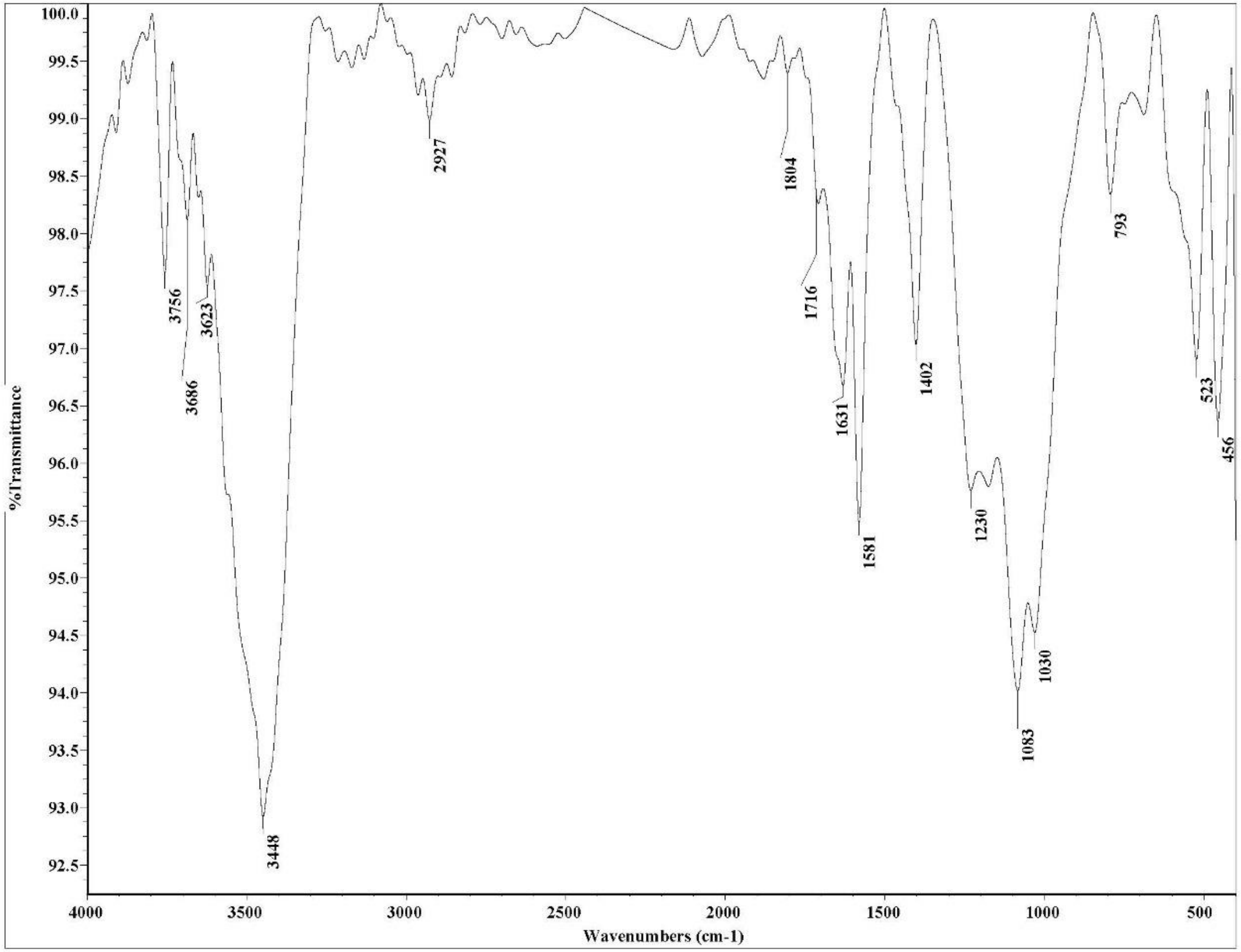

Figure S7. FTIR spectrum for pure carbon nanotubes (CNTs).

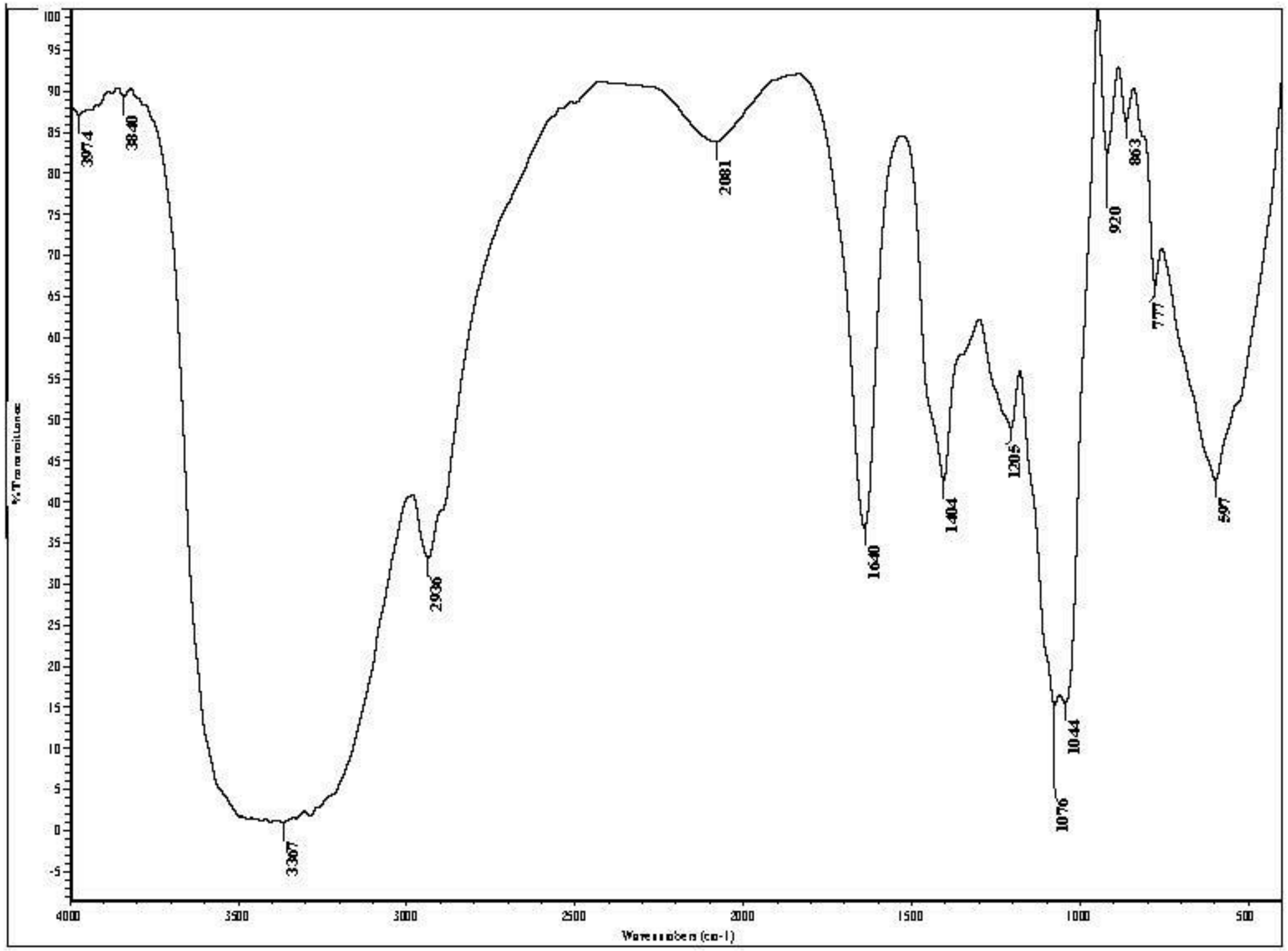

Figure S8. FTIR spectrum for CNTs loaded with nystatin.

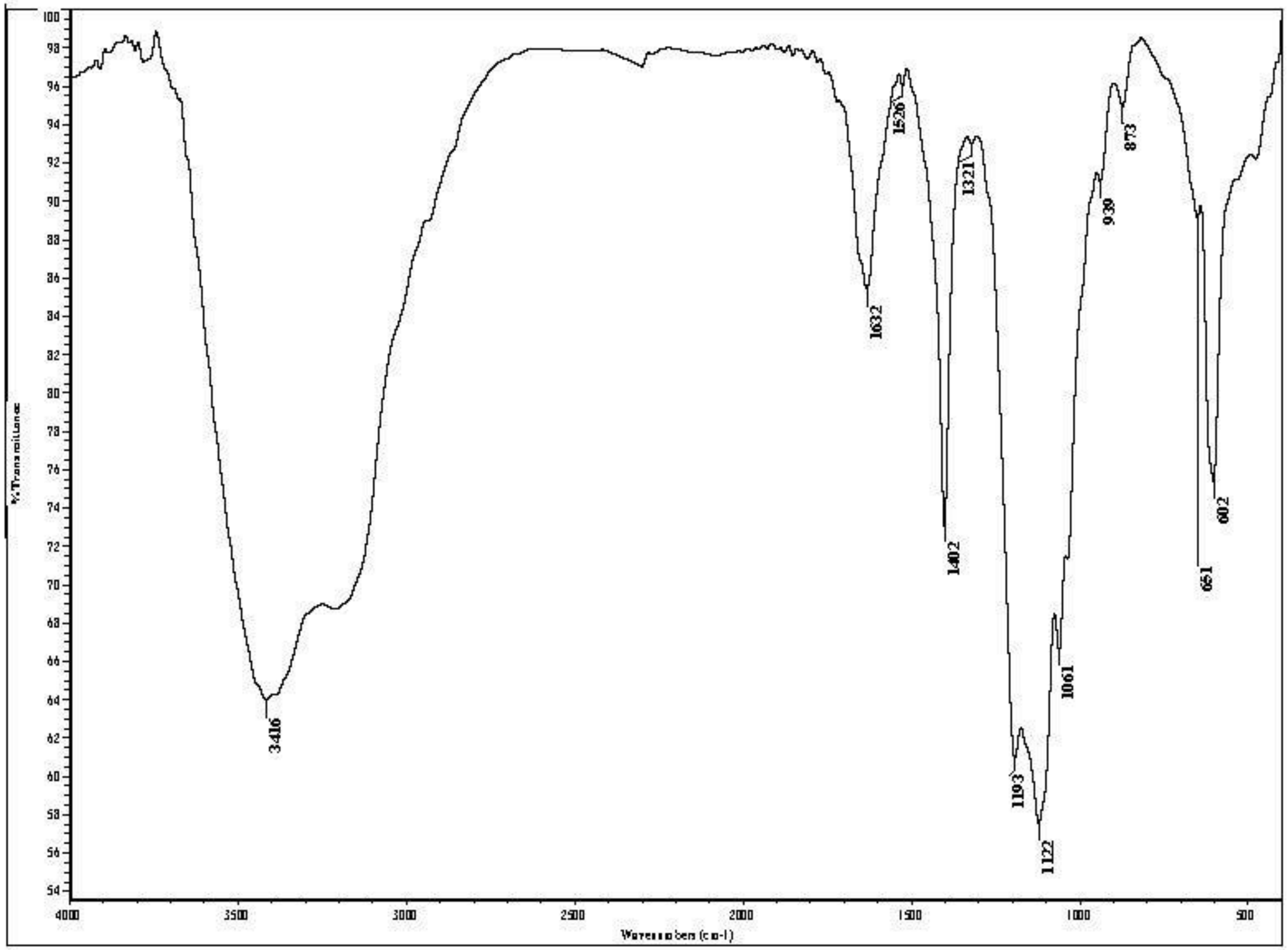

Figure S9. FTIR spectrum for CNTs loaded with fluconazole.
